# Corticolimbic hyper-response to emotion and glutamatergic function in people with high schizotypy: a multimodal fMRI-MRS study

**DOI:** 10.1038/tp.2017.53

**Published:** 2017-04-04

**Authors:** G Modinos, A McLaughlin, A Egerton, K McMullen, V Kumari, G J Barker, C Keysers, S C R Williams

**Affiliations:** 1 Institute of Psychiatry, Psychology and Neuroscience, King's College London, London, UK; 2 Centre for Brain Health, University of British Columbia, Vancouver, BC, Canada; 3 Social Brain Laboratory, Netherlands Institute for Neuroscience, Netherlands Academy for Arts and Sciences (KNAW), Amsterdam, The Netherlands; 4 Department of Psychology, University of Amsterdam, Amsterdam, The Netherlands

## Abstract

Animal models and human neuroimaging studies suggest that altered levels of glutamatergic metabolites within a corticolimbic circuit have a major role in the pathophysiology of schizophrenia. Rodent models propose that prefrontal glutamate dysfunction could lead to amygdala hyper-response to environmental stress and underlie hippocampal overdrive in schizophrenia. Here we determine whether changes in brain glutamate are present in individuals with high schizotypy (HS), which refers to the presence of schizophrenia-like characteristics in healthy individuals, and whether glutamate levels are related to altered corticolimbic response to emotion. Twenty-one healthy HS subjects and 22 healthy subjects with low schizotypy (LS) were selected based on their Oxford and Liverpool Inventory of Feelings and Experiences rating. Glutamate levels were measured in the anterior cingulate cortex (ACC) using proton magnetic resonance spectroscopy, followed by a functional magnetic resonance imaging (fMRI) scan to measure corticolimbic response during emotional processing. fMRI results and fMRI × glutamate interactions were considered significant after voxel-wise *P*<0.05 family-wise error correction. While viewing emotional pictures, HS individuals showed greater activation than did subjects with LS in the caudate, and marginally in the ACC, hippocampus, medial prefrontal cortex (MPFC) and putamen. Although no between-group differences were found in glutamate concentrations, within the HS group ACC glutamate was negatively correlated with striatal activation (left: *z*=4.30, *P*=0.004 and right: *z*=4.12 *P*=0.008 caudate; left putamen: *z*=3.89, *P*=0.018) and marginally with MPFC (*z*=3.55, *P*=0.052) and amygdala (left: *z*=2.88, *P*=0.062; right: *z*=2.79, *P*=0.079), correlations that were not present in LS subjects. These findings provide, to our knowledge, the first evidence that brain glutamate levels are associated with hyper-responsivity in brain regions thought to be critical in the pathophysiology of psychosis.

## Introduction

Compelling support has recently accumulated for a continuum model of psychosis.^1^ With mounting evidence from clinical, genetic, neurobiological, social and environmental studies, this view proposes a dimensional continuity between subclinical psychotic experiences in healthy individuals (also termed *schizotypy*) and clinically relevant psychosis.^2, 3^ An underlying factor structure that broadly corresponds to the positive, negative and disorganized dimensions of schizophrenia is commonly found for subclinical psychotic experiences,^3, 4, 5^ and recent reviews acknowledge the multidimensionality of the schizotypy construct, proposing its use as a broad label subsuming positive, negative and disorganized facets.^6, 7^ While the majority of healthy people with schizotypy as identified through self-report questionnaires are not expected to develop psychosis, high schizotypy (HS) is associated with higher risk for developing a psychotic disorder,^8^ and represents a useful and widely applied paradigm to investigate etiological factors of schizophrenia spectrum disorders.^6^ Consistent with the continuum model of psychosis, individuals with HS scores demonstrate similar—albeit attenuated—abnormalities in the processing of social and emotional information to patients with schizophrenia.^9^ Socio-emotional dysfunctions are some of the most commonly observed symptoms in schizophrenia, have a negative impact on social and vocational function, are associated with poor outcomes and are not effectively treated by available antipsychotic medications.^10, 11^

Emotional and social dysfunctions in schizophrenia involve impairments in emotional perception and expression, as well as heightened emotional responsivity and arousal.^12, 13^ Such dysfunctions have measurable neural correlates, with functional magnetic resonance imaging (fMRI) studies consistently demonstrating abnormalities within a corticolimbic network including the prefrontal cortex and anterior cingulate cortex (ACC), insula, amygdala, hippocampus and striatum.^9, 14, 15^ These findings converge with animal and post-mortem evidence, suggesting that dysregulated corticolimbic interactions play an important role in the development of schizophrenia-like characteristics.^16, 17, 18^ In rodent models of psychosis, increased medial prefrontal levels of the excitatory neurotransmitter glutamate (Glu), due to a reduction in GABAergic inhibition of local pyramidal neurons, are proposed to lead to amygdala hyper-responsivity to environmental stress.^19, 20^ Amygdala hyper-responsivity is found to reduce GABAergic interneuron function in the hippocampus through direct projections, leading to disinhibition of pyramidal cells and consequently elevating hippocampal activity.^21^ In turn, heightened output from the hippocampus to the striatum is shown to drive the striatal dopamine dysregulation that is characteristic of schizophrenia.^22, 23^ In human studies, hyper-responsivity of the amygdala and related emotional regions is observed in patients with schizophrenia^24, 25, 26^ and individuals at ultra-high risk (UHR) for psychosis,^27, 28^ as well as recently in the largest population-based study of emotional processing in healthy people with subclinical psychotic experiences.^29^ In separate research, increased Glu concentrations are found in schizophrenia across several corticolimbic areas,^30, 31^ and prefrontal elevations in Glx (glutamate+glutamine) levels in antipsychotic-naive individuals at UHR for psychosis,^31^ thus highlighting a role for abnormal levels of glutamatergic metabolites in increasing psychosis vulnerability. However, despite animal models suggesting an association between prefrontal glutamatergic neurotransmission and corticolimbic function, the interactions between these abnormalities in man remains unclear.

We investigated this issue by examining corticolimbic response during emotional processing and its relationship with regional Glu levels in a sample of healthy individuals with HS, relative to similar individuals with low schizotypy (LS) scores. While such subjects do not have clinical symptoms of psychosis, they allow investigation of processes on the psychosis continuum to be studied without the potentially confounding effects of previous antipsychotic treatment or illness chronicity. Unlike the UHR research paradigm, which designates help-seeking individuals showing attenuated clinical signs of psychosis who are in the putative prodrome of a psychotic illness (as determined with clinical diagnostic interviews such as the Comprehensive Assessment of At Risk Mental States^32^), the HS paradigm involves typically non-treatment-seeking individuals from the general population who show high levels of schizotypy or subclinical psychotic experiences, commonly identified through psychometrically validated self-report measures (for example, the Oxford and Liverpool Inventory of Feelings and Experiences (O-LIFE) questionnaire,^33^ or the Schizotypal Personality Questionnaire (SPQ)^4^). On the basis of evidence for corticolimbic dysfunction during emotional processing in healthy people with subclinical psychotic experiences,^29, 34, 35^ largely convergent with reports of structural abnormalities in overlapping regions in such individuals,^36, 37, 38^ and the above-mentioned magnetic resonance spectroscopy (MRS) findings in schizophrenia and UHR studies, we hypothesized that, relative to those with LS, HS subjects would show (1) corticolimbic circuit hyper-reactivity to emotional stimuli, (2) increased Glu levels in the ACC and that (3) interactions between these two measures would be altered in subjects with HS.

## Materials and methods

### Participants

Healthy participants were included in the study based on their score on the short version of the O-LIFE questionnaire.^33^ To capture the extremes of the distribution and have a balanced proportion of participants with high and low O-LIFE scores, 250 subjects who responded to online advertisement (Research Volunteer Recruitment Webpage of King's College London, KCL) were pre-screened. As in previous imaging research in HS,^34^ we invited participants with high levels of unusual experiences (HS group; that is, scored >7 on the Unusual Experiences (UE) subscale of the O-LIFE), and participants with a low level of UE (LS group; that is, scored <2 on the O-LIFE UE subscale). The UE subscale of the O-LIFE was chosen because it is associated with higher severity of positive symptoms in patients with schizophrenia.^39^

The recruited sample included 23 individuals in the HS group (11 males; age range, 18–55 years; mean age 28.48 years) and 25 in the LS group (14 males; age range, 18–58 years; mean age 28.36 years). Participants did not have any personal or first-degree family history of neurologic or psychiatric disorders, as confirmed both with the Mini International Neuropsychiatric Inventory^40^ (administered by a trained interviewer and reviewed by an experienced neuropsychologist (G Mosinos)) and the Psychosis Screening Questionnaire.^41^ Participants had not used recreational drugs in the 2 weeks prior to MRI scanning, and did not meet criteria for substance dependency by self-report. Ethical approval for the study was obtained through the KCL Research Ethics Committee system and all participants provided written informed consent.

### Behavioral measures

Before the start of the scanning session, all subjects completed the following assessments: the SPQ^4^ as additional measure of schizotypal symptoms; a semi-structured interview adapted from the Early Psychosis Prevention and Intervention Centre (EPPIC) Drug and Alcohol Assessment Schedule (http://www.eppic.org.au) to assess current/past medication use and current/past use of alcohol, tobacco and illicit drugs; the Social Functioning Questionnaire (SFQ)^42^ to measure social functioning; and a validated short version of the Wechsler Adult Intelligence Scale-III (WAIS-III)^43^ to measure intelligence.

### fMRI task

The fMRI task was the same as in a previous study from our group in patients with first-episode psychosis and UHR subjects.^27^ The stimulus set consisted of 50 color pictures from the International Affective Picture System,^44^ 10 in each of the following categories: negative high arousal (NHA), negative low arousal (NLA), positive high arousal (PHA), positive low arousal (PLA) and neutral (NEU), matched for social content (~50%). Pictures for each category were chosen based on normative ratings,^44, 45^ and the final selection of images was the same as in our previous study.^27^ Before scanning, all subjects were trained on the task using 10 International Affective Picture System images different from those used in the fMRI experiment.

Participants were scanned while viewing each picture for 4000 ms, followed by a gray screen showing a fixation cross that served as low-level baseline condition (varying from 1000 to 10 000 ms). This was followed by a rating screen presented for 4000 ms during which time subjects indicated their subjective emotional arousal to the previously presented stimulus via button press (1=not at all aroused, 2=slightly aroused, 3=highly aroused). Arousal ratings and reaction times were used as behavioral metrics of emotional processing. Trial presentation order was pseudo-randomized based on simulations to optimize experimental power.

### fMRI acquisition and preprocessing

Echo-planar images sensitive to blood oxygenation level-dependent (BOLD) contrast were acquired to measure hemodynamic responses on a General Electric Discovery MR750 3 T system (Milwaukee, WI, USA) at the Institute of Psychiatry, Psychology and Neuroscience, King's College London (repetition time: 2000 ms; echo time, 30 ms; flip angle, 75° 3.3 × 3.3 × 3.0-mm voxels; field of view, 211; 41 axial sections collected with sequential (top down) acquisition and 0.3-mm interslice gap). Structural data were acquired by means of a three-dimensional T1-weighted inversion recovery-prepared gradient echo sequence (voxel size: 1.05 × 1.05 × 1.2 mm, field of view: 270 mm, 196 slices, repetition time: 7.3 ms, echo time: 3.0 ms, inversion time: 400 ms). Four participants had to be excluded because of failure to complete the fMRI task (3 LS, 1 HS).

Functional MRI data were preprocessed using the SPM12 software (http://www.fil.ion.ucl.ac.uk/spm/software/spm12). After slice timing, realignment, segmentation, co-registration and stereotaxic normalization (2 × 2 × 2 mm^3^), images were spatially smoothed using an 8-mm full-width at half-maximum Gaussian filter and a high pass filter (128 s). Excessive movement was considered at >3 mm of translation and 3 degree of rotation in any axis; no images exceeded this threshold.

### MRS acquisition and quantification

**Figure 1:**
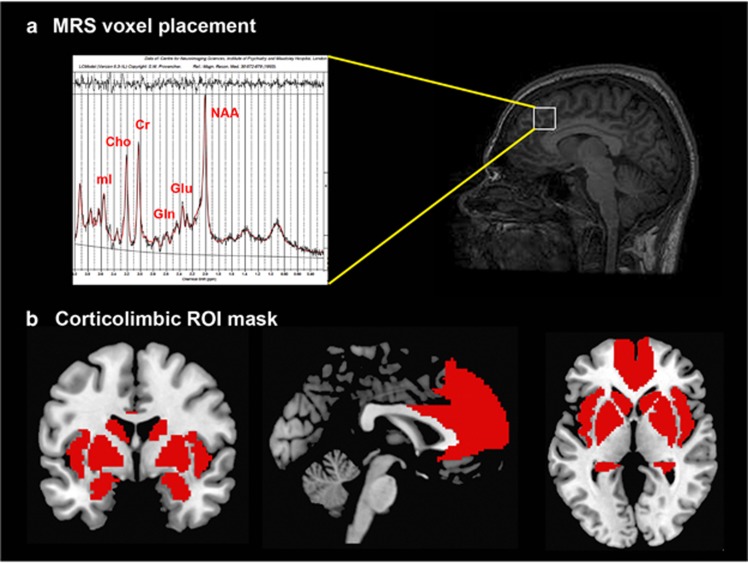
(**a**) Magnetic resonance spectroscopy (MRS) voxel placement in the anterior cingulate cortex. (**b**) Pre-defined anatomical mask used for region of interest (ROI) analysis within an emotional processing circuit.

A proton MRS (^1^H-MRS; PRESS, Point RESolved Spectroscopy) was acquired during the same scanning session, prior to the fMRI task, from the ACC (Figure 1a), as described in previous studies from our group in UHR subjects and patients with first-episode psychosis.^46, 47, 48^ Total scanning time was ~75 min.

A standard GE PROBE (proton brain examination) sequence was used, which incorporates a standardized chemically selective suppression (CHESS) water suppression routine (echo time: 30 ms; repetition time: 3000 ms; 96 averages were collected). For each acquisition, unsuppressed water reference spectra (16 averages) were also acquired. Shimming and water suppression were optimized, with auto-prescan performed twice before each scan. The region of interest (ROI) in the ACC was prescribed from the midline sagittal localizer, and the center of the 20 × 20 × 20 mm ROI was placed 13 mm above the anterior section of the genu of corpus callosum at 90° to the anterior comissure - posterior comissure line.

Spectra were analyzed using LCModel version 6.3-1l (http://s-provencher.com/pages/lcmodel.shtml).^49^ Water-scaled Glu, glutamine (Gln), Glx, myo-inositol, choline, creatine and *N*-acetylaspartate values were corrected for the cerebrospinal fluid (CSF) content of the voxel using the formula: metabolite corrected=metabolite concentration × (proportion WM+proportion GM+(1.55 × proportion CSF))/(proportion WM+proportion GM), where GM (gray matter) and WM (white matter). We determined the voxel CSF content for each subject by extracting the location of the voxel from the spectra file headers, and using an in-house program to calculate the percentage GM, WM and CSF content using the segmented T1-weighted images, to correct the spectroscopy results for partial volume CSF contamination. Cramer–Rao minimum variance bounds>20% as reported by LCModel, which are estimates of fit of the metabolite peaks, was used to determine poorly fitted metabolite peaks for exclusion from statistical analysis;^50^ one subject exceeded this threshold and the final sample thus involved 21 HS subjects and 22 LS subjects (in line with previous imaging studies detecting significant effects in HS during social cognitive tasks^35, 51, 52^). The primary ^1^H-MRS measure was Glu corrected for voxel CSF.

### Statistical analysis

#### Behavioral data

Analysis of behavioral and demographic data was performed in SPSS 23 (http://www-01.ibm.com/software/uk/analytics/spss/). The effect of group on these measures was tested using independent sample *t-*tests for parametric data and *X*^2^-tests for non-parametric data. Between-group differences in emotional processing were tested using a repeated-measures analysis of variance with ‘Condition' as within-subject factor (NHA, NLA, PHA, PLA and NEU) and ‘Group' (LS, HS) as between-subject factor. The same procedure was used for analysis of reaction time. Significant effects are reported at *P*<0.05 and trend effects at *P*<0.1.

#### fMRI analysis

Statistical analyses of fMRI data were conducted using the general linear model implemented in SPM12. Separate regressors of interest were specified for each trial type: NHA, NLA, PHA, PLA and NEU. In addition, realignment parameters (*x*, *y*, *z*, pitch, roll, yaw) were included in all first-level models as covariates of no interest to account for variance associated with head movement. All regressors were convolved with a canonical hemodynamic response function during the 4000 ms ‘viewing' screen, in order to focus on activation related to experiencing emotion rather than to cognitively assessing one's subjective response to stimuli.^27^

One contrast image was generated for each participant examining emotional-related activation, by contrasting all emotional trials versus neutral trials, which was then submitted to an independent samples *t*-test for second-level analysis in SPM12. Emotional perception comprises a network of regions involved in core affect processing, including the medial prefrontal cortex (MPFC) and ACC, the insula, medial temporal regions (hippocampus, amygdala) and the striatum (caudate, putamen, pallidum).^53, 54^ Thus, we restricted our analyses to this circuitry using a ROI approach, with a mask created with automated anatomical labeling as implemented in the WFU_Pickatlas toolbox in SPM (Figure 1b shows our ROI mask overlaid on a standard brain template). We used an initial search threshold of *P*<0.005 uncorrected, to then enforce voxel-wise correction for multiple testing at *P*<0.05 family-wise error.

#### ^1^H-MRS analysis

Between-group differences in ACC Glu concentrations were examined with an independent samples *t*-test in SPSS. Exploratory analyses of the other metabolites present in the spectra were also analyzed with a *t*-test corrected for multiple comparisons (threshold for six metabolites, one voxel; *P*=0.008). Levene's test was used to check for equality of variance across groups.

#### Integration of fMRI and ^1^H-MRS data

The relationship between the BOLD response to emotional scenes within our network of interest and Glu levels in the ACC was investigated by entering the individual Glu values as covariates in an analysis of variance design with the fMRI contrast images (emotional>neutral) using SPM12. Glutamate × BOLD response interactions were assessed separately for subjects with LS and subjects with HS, to then interrogate group × glutamate × BOLD response interactions in the same SPM design matrix. As above, an initial search threshold was set at *P*<0.005 uncorrected, to then consider significant regions surviving voxel-wise correction at *P*<0.05 family-wise error.

## Results

Full demographic and behavioral results are presented in Table 1. Briefly, the only significant differences between our study groups related to the schizotypy measures. Individuals with HS showed higher scores than those with LS on O-LIFE: total (*P*<0.001), UE (*P*<0.001) and cognitive disorganization (*P*=0.001). We also observed significantly higher levels of SPQ: total (*P*<0.001), suspiciousness (*P*=0.001), ideas of reference (*P*<0.001), odd speech (*P*=0.005), odd beliefs (*P*=0.002), and unusual perceptual experiences (*P*<0.001) in HS compared to LS subjects.

### Behavioral performance

There was a main effect of condition in arousal ratings (*F*(4,3.231)=105.776, *P*<0.001), by which NHA pictures were rated as most emotionally arousing compared to all other conditions, followed by PHA, PLA, NLA. NEU pictures were rated as least arousing, and there was no difference between NLA and PLA ratings. There was no evidence for a main effect of group (*F*(1,41)=1.739, *P*=0.195) or group × condition interaction (*F*(4,3.231)=1.890, *P*=0.130). For reaction time, there was a trend for a significant main effect of condition (*F*(4,1.000)=2.408, *P*=0.051), but no main effect of group (*F*(1,41)<1, n.s.), or group × condition interaction (*F*(1,41)<1, n.s.).

### fMRI results

**Figure 2:**
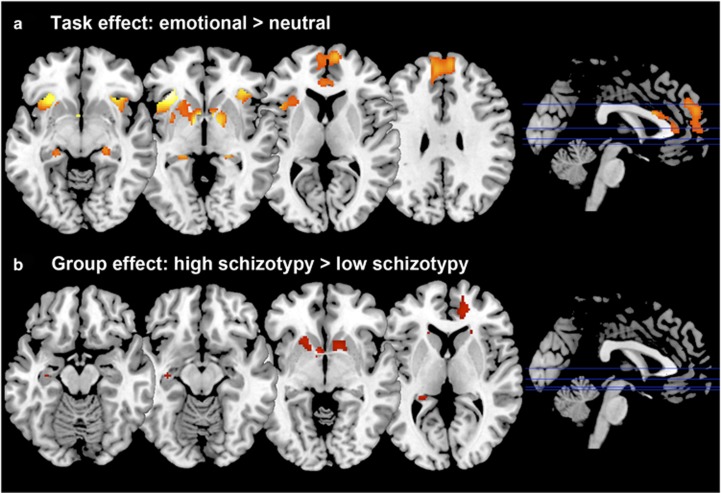
(**a**) Statistical parametric maps showing activation during the emotional processing task across all subjects (Emotional>Neutral). (**b**) Brain areas where high schizotypy (HS) subjects showed greater activation than those with low schizotypy (LS). All effects considered significant at voxel-wise *P*<0.05 family-wise error correction; statistical parametric maps thresholded at *P*<0.005 uncorrected for display purposes.

As expected, across groups the contrast Emotional>Neutral induced increased activation in the MPFC, ACC and bilaterally in the striatum, insula, hippocampus and amygdala (Table 2 and Figure 2a).

Group comparisons revealed hyper-responsivity to emotional pictures in HS subjects compared to LS subjects in the caudate, and trend-level hyper-responsivity in the ACC, hippocampus, MPFC and putamen (Table 2 Figure 2b). There were no areas of significantly lower activation in HS relative to LS individuals.

### ^1^H-MRS spectral quality

Spectra obtained were of good quality, with LCModel reporting mean±SD signal-to-noise ratio of 25.44±4.5 and line width of 4.83±0.8 Hz. For Gln, only data from 9 LS subjects and 11 HS subjects were usable (Cramer–Rao minimum variance bounds<20%). There were no significant group differences in any of the parameters relating to spectral quality or in voxel tissue content. Data relating to spectral quality by group are presented in Table 3.

### ^1^H-MRS results

No between-group differences were found in Glu concentrations, or in any of the other metabolites that could be reliably quantified within the voxel selected (Gln, Glx, myo-inositol, choline, creatine and *N*-acetylaspartate; Table 3). There were no significant correlations between metabolite concentrations and age.

### fMRI group x glutamate interactions

**Figure 3:**
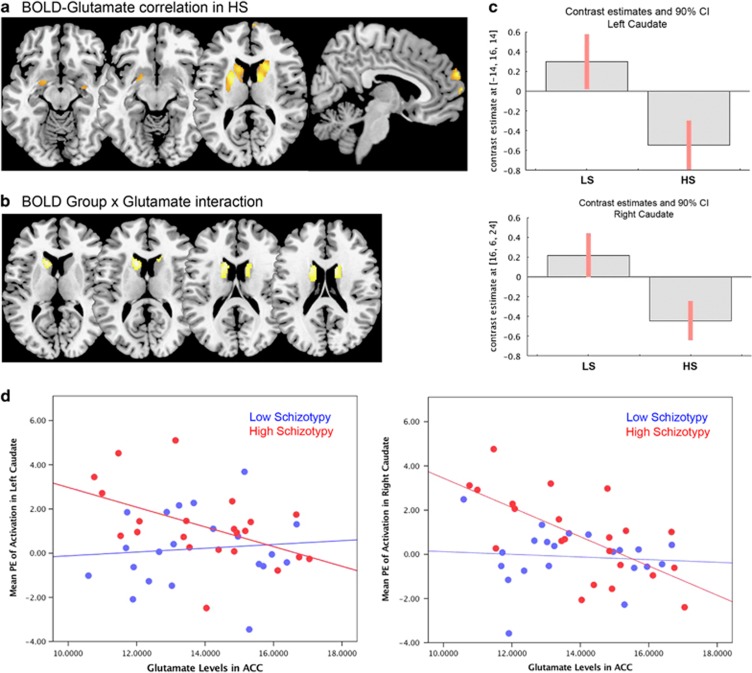
(**a**) Section views of the negative association between glutamate levels in the anterior cingulate cortex (ACC) and functional activation during emotional processing in high schizotypy (HS) subjects. (**b**) Section views of the interaction between Group activation to emotion and ACC glutamate levels. (**c**) Plots showing the interaction between left and right caudate activation and glutamate levels in the HS group relative to low schizotypy (LS). (**d**) Scatterplots of the association between ACC glutamate levels and activation in the left and right caudate. All effects considered significant at voxel-wise *P*<0.05 family-wise error (FWE) correction; statistical parametric maps shown at *P*<0.005 uncorrected for display purposes. BOLD, blood-oxygenation level dependent.

Within the HS group, there was a significant negative correlation between ACC Glu levels and response to emotional stimuli in a striatal region spanning adjacent parts of the left caudate and putamen, as well as in the right caudate, and at trend level in the MPFC and in the amygdala bilaterally (Table 4 and Figure 3a). Furthermore, there was a significant interaction between ACC Glu levels, BOLD response to emotion and Group in the caudate bilaterally, which was driven by the negative association in the HS group compared to LS (Table 4; Figure 3b).

## Discussion

The main finding of our study is that individuals with HS show hyper-reactivity to emotional pictures in the striatum, and marginally in the hippocampus, ACC and MPFC compared to those with LS. Although there were no significant differences in ACC Glu concentrations between the two groups, in HS subjects Glu levels were negatively associated with the degree of activation to emotional pictures in the striatum, as well as marginally in the amygdala and MPFC. These associations were absent in the LS group. This may suggest that hyperactive neural responses during emotional processing in schizotypal individuals are related to decreased ACC Glu concentrations.

Hyper-responsivity within an emotional processing circuitry has been described in patients with schizophrenia and individuals at UHR for psychosis.^24, 25, 27, 28, 55^ The principal regions within this circuitry are the MPFC (including the ACC), anterior insula and limbic areas such as the amygdala, hippocampus and striatum.^53^ This circuitry instantiates neural representations of core affect, and provides the substrate for perception and experience of emotion. These neural representations indicate whether an object or situation is helpful or harmful, rewarding or threatening, requiring acceptance or rejection.^54^ Core affect is underpinned by amygdala recruitment relating to the salience or potential information value of stimuli,^56, 57^ with the striatum (caudate, putamen and globus pallidus) assessing the affective value of stimuli in a more general sense across motivation, reward and learning,^53^ the anterior insula assessing interoceptive cues^58^ and prefrontal regions contributing to making inferences about one's own moment-to-moment feelings and engaging in emotional regulation processes.^53^ In turn, this circuitry projects to midbrain and brainstem areas that influence autonomic, chemical and behavioral responses to help establish an affective representation of an object.^54^ Therefore, the involvement of this corticolimbic circuitry is not limited to entailing a pleasant or unpleasant feeling but it also controls the degree of cortical and physiological arousal to a given stimulus. In an independent group of healthy subjects with HS, we previously reported heightened neural response and decreased functional connectivity during the regulation of negative emotion.^35^ We also previously showed that a differential pattern of activation within the emotional brain circuitry could accurately classify individuals with HS versus those with LS.^51^ In addition, further support for neural hyper-response to emotion in HS has been recently provided in the largest community sample examined to date.^29^ It is noteworthy that structural MRI studies in healthy subjects with HS have reported converging abnormalities in similar regions. For example, gray matter volume/density reductions in HS compared with LS subjects have been reported in MPFC, orbitofrontal and temporal regions,^37^ in the insula and dorsolateral PFC,^38^ as well as negative correlations between MPFC volume and schizotypal personality measures.^59^ In addition, gray matter volume increases in HS compared with LS individuals have been found in posterior cingulate cortex and precuneus,^60^ as well as positive correlations between cortical thickness in dorsolateral PFC and SPQ total score,^61^ and between gray matter volume in the precuneus and negative-dimension schizotypy.^36^ Collectively, these findings support the view that subclinical psychotic experiences in healthy subjects and psychotic symptoms in patients with schizophrenia share similar neurobiological bases, and that dysfunction of the circuitry underlying emotional processing has an important role in the expression of psychotic-like experiences.

The present study did not find direct differences in Glu levels in subjects with HS compared to those with LS. The first meta-analytic effort at synthesizing evidence of regional Glu concentrations in patients with schizophrenia indicated converging reductions in MPFC/ACC Glu levels in schizophrenia, while Gln levels were found to be increased compared with controls.^30^ A more recent meta-analysis included measurements in other brain regions as well as in subjects at UHR for psychosis and with a first-episode of psychosis, allowing putative effects of disease stage and exposure to antipsychotics to be ascertained.^31^ Elevated medial frontal Glx levels were observed in UHR individuals, which were absent after illness onset (first-episode psychosis and chronic schizophrenia). In contrast, elevated Glx in the medial temporal lobe was found in chronic schizophrenia but not in early psychosis (UHR or first-episode psychosis groups).^31^ In schizotypy, the only previous study using MRS reported no significant correlation between Glx levels in the thalamus and scores on a measure of magical ideation.^62^ Similarly, we did not find significant group differences in any of the other measurable metabolites (including Glx) in our prefrontal voxel. These findings suggest that sizeable regional alterations in MPFC/ACC glutamatergic metabolites may appear at a later stage, once psychotic-like experiences are exacerbated and the phenotype becomes clinically relevant. As our report is, to our knowledge, the first to interrogate prefrontal glutamatergic function in HS, future studies are warranted to replicate our findings.

Our third prediction was that the relationship between neurophysiological and neurochemical measures would be altered in subjects with HS. This hypothesis was confirmed. Within the HS group, functional activation in the emotional circuitry was negatively correlated with Glu levels in the ACC, whereas in LS these associations were lacking. Recent studies in the healthy brain reported a positive correlation between prefrontal Glu levels and ACC activation to facial expressions of anger,^63^ as well as positive correlations between ACC Glu levels and local response during an empathy task.^64^ Our finding of an inverse correlation between Glu levels and functional response to emotion in HS individuals was in the opposite direction and significantly different from that observed in the LS group. In UHR individuals, multimodal studies have also demonstrated negative associations between thalamic Glu levels and prefrontostriatal activation during verbal fluency tasks,^65, 66^ as well as between temporal Glu levels and local activation during verbal encoding.^67^ A direct relationship between corticolimbic hyper-responsivity to emotion and Glu levels in HS provides support to the notion that dysfunction of this circuitry may have an important role in the pathophysiology of psychotic-like characteristics, as suggested by converging animal and human evidence. More specifically, a validated neurodevelopmental preclinical model of psychosis proposes that an imbalance of Glu and GABAergic function in prefrontal brain regions would induce a failure to regulate the amygdala response to emotion, thereby unfolding a neurobiological cascade to striatal hyperdopaminergia via excitation/inhibition imbalances through a multisynaptic pathway including the hippocampus,^68^ leading to schizophrenia-like symptoms.^23^ The critical role of emotional dysregulation and excitation/inhibition imbalances has been further illustrated by recent work, showing that premorbid administration of benzodiazepines at anxiolytic doses in an animal model prevents the emergence of psychotic-like characteristics in adulthood (such as hyperdopaminergia, hippocampal hyperactivity, increased locomotor response to amphetamine).^16, 69^ Future longitudinal studies testing this model comprehensively, including subjective and neurophysiological responses to emotion, and their association with GABAergic, glutamatergic and dopaminergic neurotransmission, will be fundamental to understanding the mechanisms underlying the development of schizophrenia, and may provide a scientific basis for the development of novel interventions focused on emotional regulation to prevent or delay progression from the vulnerability to the psychotic state.

While patients with schizophrenia and UHR subjects report stronger subjective feelings of emotion than healthy controls,^9, 13^ we did not observe an increase in subjective arousal in HS compared with LS subjects. The lack of significant group effects on subjective emotion could indicate that experiential correlates of corticolimbic dysfunction may become manifest at stages in the psychosis continuum that are closer to a clinically relevant disorder. An alternative explanation may be that the observed activation increases reflect compensatory mechanisms in these otherwise healthy, non help-seeking individuals. In fact, we observed negative interactions between ACC Glu and fMRI response to emotion, suggesting that the observed hyperactivation during emotional processing in schizotypal individuals is related to lower ACC Glu levels. Although in schizophrenia and UHR the relationship between prefrontal Glu or Glx levels (typically increased) and functional response to emotion (typically increased) is yet to be investigated, our study in HS suggests opposite fMRI-MRS correlations from what would be predicted in those clinical groups. As our study involves high-functioning HS individuals, our findings may provide evidence for potentially protective neurobiological mechanisms in this population. Further research examining the relative contributions of excitatory and inhibitory neurotransmission in these different groups (schizophrenia, UHR, HS) and their interactions with functional response during emotional tasks has the potential to provide substantial insights into the neurobiology of risk and resilience for psychiatric disorders.^70^

As a limitation to our study, the results should be considered in the context of subjects pooled from a university sample, with relatively high IQs and no differences in substance use between groups, and as such may not generalize to all individuals with schizotypy. Larger community-based studies would help define the normal variation in schizotypy. In addition, MRS concentration estimates reflect both intra- and extracellular Glu and are consequently unable to discriminate between neuronal (e.g., pyramidal) and non-neuronal (that is, glia) metabolite concentrations; therefore, the present results should be interpreted to reflect total tissue Glu levels rather than glutamatergic neurotransmission specifically. Finally, using our approach of PRESS with an echo time of 30 ms at  3 Tesla, the partially overlapping signals from Glu and Gln cannot be entirely resolved, with contamination of the Glu signal by Gln estimated as <10%.^71^

In summary, the present study suggests that hyper-responsivity within a neural circuitry underlying emotional processing is associated with Glu levels in subjects with HS. These findings support the view that there is neurobiological continuity between subclinical psychotic experiences in healthy individuals and psychotic experiences in schizophrenia, while also indicating potential neurobiological mechanisms of resilience, which may be at play in schizotypy. Future multimodal studies investigating the pathway linking emotional dysregulation and the neurotransmitter systems GABA and Glu in different groups along the psychosis continuum have the potential to unveil a mechanistic framework for the development of psychosis, and to demonstrate whether clinical interventions targeting this pathway have the potential to block the development of psychosis in vulnerable individuals.

## Figures and Tables

**Table 1:** Sample characteristics

	*Low schizotypy (*n=*21)*	*High schizotypy (*n=*22)*	*Analysis*
Age (years)	27.00 (5.64)	27.36 (7.61)	*t*=−0.177, *P*=0.860
Gender (% female)	42.9% (9 F)	50% (11 F)	*X*^2^=0.220, *P*=0.763
Ethnicity (% Caucasian)	76.2% (*n*=16)	59.1% (*n*=13)	*X*^2^=4.289 *, P*=0.232
IQ (WAIS-III short version)	121.10 (12.59)	117.41 (18.55)	*t*=0.052, *P*=0.452
			
*O-LIFE total*	16.24 (8.92)	38.23 (12.62)	*t*=−6.620, *P*<0.001
O-LIFE unusual experiences	0.86 (1.01)	11.59 (4.93)	*t*=−10.003, *P<*0.001
O-LIFE cognitive disorganization	5.38 (4.14)	11.32 (6.69)	*t*=−3.518, *P*=0.001
O-LIFE introvertive anhedonia	4.90 (3.21)	9.05 (2.50)	*t*=−4.736, *P*<0.001
O-LIFE impulsive nonconformity	5.10 (4.35)	6.27 (4.60)	*t*=−0.863, *P*=0.393
			
*SPQ total*	9.05 (8.57)	23.89 (14.04)	*t*=−4.160, *P*<0.001
SPQ suspiciousness	0.43 (.68)	2.45 (2.32)	*t*=−3.918, *P*=0.001
SPQ constricted affect	1.14 (1.65)	1.82 (1.84)	*t*=−1.263, *P*=0.214
SPQ ideas of reference	0.19 (0.51)	3.36 (2.40)	*t*=−6.056, *P*<0.001
SPQ no close friends	1.43 (2.06)	2.41 (2.04)	*t*=−1.567, *P*=0.125
SPQ odd speech	1.86 (2.08)	3.91 (2.51)	*t*=−2.955, *P*=0.005
SPQ odd/eccentric behavior	1.33 (2.01)	2.45 (2.41)	*t*=−1.655, *P*=0.106
SPQ odd beliefs	0.05 (0.22)	1.41 (1.84)	*t*=−3.440, *P*=0.002
SPQ excessive social anxiety	2.33 (2.08)	3.30 (2.58)	*t*=−1.343, *P*=0.187
SPQ unusual perceptual experiences	0.29 (0.46)	2.73 (2.38)	*t*=−4.730, *P*<0.001
			
Social functioning questionnaire total	4.10 (3.13)	5.50 (2.87)	*t*=−1.534*, P*=0.133
Daily tobacco use (mean)	0.78 (3.35)	0.30 (0.75)	*t*=0.619, *P*=0.540
Daily caffeine use (mean)	1.82 (1.52)	2.82 (2.52)	*t*=−1.566, * P*=0.125
Alcohol use (median (range))	2 (0–5)	1 (0–5)	*X*^2^=5.046, * P*=0.410
Marijuana use (median (range))	1 (0–3)	0 (0–3)	*X*^2^=2.562, * P*=0.464
Parental socio-economic status (% professional level)	66.7% (*n*=14)	63.6% (*n*=14)	*X*^2^=0.343, * P*=0.842
Educational level (% university level)	90.5% (*n*=19)	77.3% (*n*=17)	*X*^2^=1.374, * P*=0.241

Abbreviations: O-LIFE, Oxford–Liverpool Inventory of Feelings and Experiences; SPQ, Schizotypal Personality Questionnaire; WAIS-III, Wechsler Adult Intelligence Scale-III.

**Table 2:** Foci of activation during the emotional processing task (Emotional>Neutral contrast; region of interest analysis)

*Brain region*	*Side*	*MNI*			*Number of voxels*	Z*-score*	P*-value FWE*
		x	y	z			
*Task effects*							
Insula	L	−36	24	0	568	5.04	<0.001
	R	30	22	−12	314	4.47	0.002
Caudate	L	−2	6	−4	268	4.89	<0.001
Pallidum		−8	2	−4		4.33	0.003
Caudate	R	4	6	−4	138	4.34	0.003
Hippocampus	R	24	−6	−18	73	4.49	0.006
	R	22	−30	−4	44	3.88	0.028
	L	−22	−30	−4	64	3.49	0.071
Amygdala	R	22	−4	−18	78	4.17	0.004
	L	−18	−4	−14	17	3.01	0.067
Medial prefrontal cortex	R	8	58	18	1179	3.67	0.035
Anterior cingulate cortex	L	−2	18	22	94	3.32	0.061
							
*Group effects*							
* HS>LS*							
Caudate	L	−6	6	−6	178	3.81	0.023
Medial prefrontal cortex	R	16	56	8	91	3.39	0.081
Putamen	R	16	12	−8	91	3.39	0.085
Anterior cingulate cortex	R	16	44	4	95	3.38	0.051
Hippocampus	L	−24	−36	4	24	3.29	0.063
*LS>HS*							
No suprathreshold effects							

Abbreviations: FWE, family-wise error correction; HS, high schizotypy; L, left; LS, low schizotypy; MNI, Montreal Neurological Institute; R, right.

*P*-value (voxel-wise) after FWE.

**Table 3:** ^1^H-MRS quality parameters and metabolite levels by group

	*Low schizotypy*		*High schizotypy*		
	*Mean*	*s.d.*	*Mean*	*s.d.*	P-*value*
SNR	25.14	4.40	25.73	4.76	0.678
Line width	4.95	0.97	4.72	0.56	0.343
Voxel CSF	0.24	0.04	0.26	0.05	0.298
Voxel GM	0.64	0.05	0.63	0.05	0.376
Voxel WM	0.11	0.03	0.11	0.04	0.830
Glutamate % CRLB	6.00	0.95	5.73	0.94	0.348
Glutamate	13.75	1.79	13.97	1.90	0.695
Glutamine % CRLB	14.44	4.07	14.73	3.50	0.869
Glutamine	6.08	1.21	5.80	1.52	0.661
Glx % CRLB	6.52	1.17	6.32	1.21	0.574
Glx	17.96	2.56	18.41	3.12	0.605
Creatine % CRLB	2.48	0.51	2.50	0.51	0.880
Creatine	13.14	4.24	13.56	4.56	0.759
Myo-inositol % CRLB	4.71	0.64	4.64	0.90	0.747
Myo-inositol	7.63	0.86	7.55	0.94	0.760
*N*-Acetyl-aspartate % CRLB	2.95	0.50	2.73	0.46	0.129
*N*-Acetyl-aspartate	12.94	1.19	13.19	1.42	0.538
Choline % CRLB	3.14	0.36	3.09	0.53	0.709
Choline	6.54	5.45	6.94	5.44	0.812

Abbreviations: CRLB, Cramer–Rao Lower Bounds; CSF, cerebrospinal fluid; Glx, glutamate+glutamine; GM, gray matter; ^1^H-MRS, proton magnetic resonance spectrum; SNR, signal-to-noise ratio; WM, white matter.

**Table 4:** Anterior cingulate cortex glutamate effects on fMRI response to emotional stimuli (Emotional>Neutral contrast; region of interest analysis)

*Brain region*	*Side*	*MNI*			*Number of voxels*	Z*-score*	P*-value FWE*
		x	y	z			
*Correlation in HS subjects*							
*Positive*							
No suprathreshold effect							
*Negative*							
Caudate	L	−20	4	20	695	4.30	0.004
Putamen	L	−20	-	10		3.89	0.018
Caudate	R	16	0	20	759	4.12	0.008
Medial prefrontal cortex	R	4	64	26	107	3.55	0.052
Amygdala	L	−10	0	−12	12	2.88	0.062
	R	28	−8	−12	8	2.79	0.079
							
*Correlation in LS subjects*							
*Positive*							
No suprathreshold effect							
*Negative*							
No suprathreshold effect							
							
*Group × ACC glutamate interaction*							
Caudate	L	−14	16	14	275	3.44	0.040
	R	16	6	24	120	3.35	0.051

Abbreviations: ACC, anterior cingulate cortex; FWE, family-wise error correction; HS, high schizotypy; L, left; LS, low schizotypy; MNI, Montreal Neurological Institute; R, right.

*P*-value after voxel-wise FWE.
